# Bilateral trans-radial approach in stenting of occluded right axillary artery

**DOI:** 10.1186/s13019-014-0138-0

**Published:** 2014-08-23

**Authors:** Sasko Kedev, Aleksandar Jovkovski, Biljana Zafirovska

**Affiliations:** Interventional Cardiology Department, University Clinic of Cardiology, Medical Faculty, University of St.Cyril & Methodius, Vodnjanska 17, Skopje, 1000 Macedonia

**Keywords:** Transradial approach (TRA), Axillary artery stenting, Chronic total occlusion (CTO), Peripheral artery disease

## Abstract

With recent advancement in percutaneous endovascular interventions, angioplasty and stenting of axillary artery lesions could become the treatment of choice vs. surgical intervention owing to its lower complication and mortality rates and shorter hospital stay.

We report a Caucasian female case with axillary artery chronic total occlusion (CTO) with dual etiology (atherosclerotic and radiation induced), which was successfully managed with stent angioplasty. The strategy used was right radial retrograde approach with contralateral injections from left radial catheter. Two year follow-up revealed widely patent axillary stents.

**Electronic supplementary material:**

The online version of this article (doi:10.1186/s13019-014-0138-0) contains supplementary material, which is available to authorized users.

## Background

There is no published data for stenting of occluded axillary artery, through the radial access. We report a case with a novel, retrograde approach for stenting of occluded right axillary artery using bilateral radial approach. The procedure was successful with patent stents after two years of follow up with color duplex-ultrasonography and angiography.

## Case presentation

Our patient is 77 years old Caucasian female with coldness and pain in the right arm during minimal physical strain and some movement inability in the same arm. The symptoms began 1 year prior to hospitalization with worsening of the symptoms 2-3 weeks ago, with augmentation of the pain in the arm that limited her everyday's home based activities. The patient had undergone duplex-ultrasonography of the upper limbs, with high suspicion of occlusion of the right axillary artery. The patient did not have prior history of trauma of the upper limbs, but had prior history of mastectomy 16 years ago treated with concomitant radiotherapy as well. From risk factors she was a smoker with hypertension and diabetes. On physical finding there was difference in systolic blood pressure readings of 50 mmHg (80 mmHg in the right brachial artery and 130 mmHg in the left brachial artery). The right radial and ulnar arteries were hardly palpable. The patient did not have any cardiac symptoms, 2D echo showed EF of 65% without enlargement of the cardiac chambers or valvular disease. All relevant laboratory examinations were within normal range. Patient was loaded with 600 mg clopidogrel and was already on 100 mg of aspirin daily before intervention. She was also pre-hydrated with 0,9% normal saline 6 hours before intervention. Written, informed consent was obtained and the procedure has been approved by the IRB of University Clinic of Cardiology, Medical Faculty in Skopje under the Presidency of Prof. Lidija Dobrkovic and members Associate Prof. Marija Vavlukis and Katerina Dimovska RN.

Coronary and peripheral angiography were performed through the left radial artery before attempting angioplasty of the occluded right axillary artery in order to exclude significant and relevant concomitant coronary artery disease.

**Figure 1:**
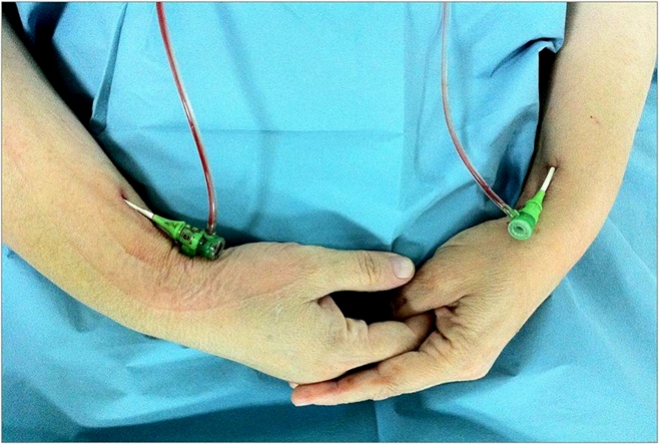
**Bilateral transradial approach with 6 F introducers.**

There was no significant coronary artery disease. Peripheral angiography with Simmons 2 5 F diagnostic catheter, (Supertorque plus, Cordis) revealed long segment of calcified CTO of the right axillary artery. We decided on using bilateral radial access and retrograde approach from right radial artery (Figure 1).

Puncturing the right radial artery due to diminished and nearly absent radial pulse was practically challenging. Radial artery was accessed after local infiltration with 1 to 1.5 ml 2% lidocaine, using counter puncture technique with a 20 G plastic iv cannula and 0.025 inch mini guide-wire of 45 cm and followed by 6Fr hydrophilic introducer sheath (Terumo, Japan) placement. Spasmolytic cocktail (5 mg verapamil) was given intra-arterially through the radial sheath. The delayed digital subtraction angiograms before procedure confirmed long chronic total occlusion of right axillary artery.

Based on the experience with long coronary CTO lesions, retrograde approach is preferred owing to the softer plaque consistency on the distal part of the CTO. During the angioplasty we needed frequent antegrade contrast injections in order to control guidewire advancement and to ensure true lumen positioning. Contralateral injections are essential in providing evidence for wiring through the true lumen and safe distal reentry of the CTO wire.

**Figure 2:**
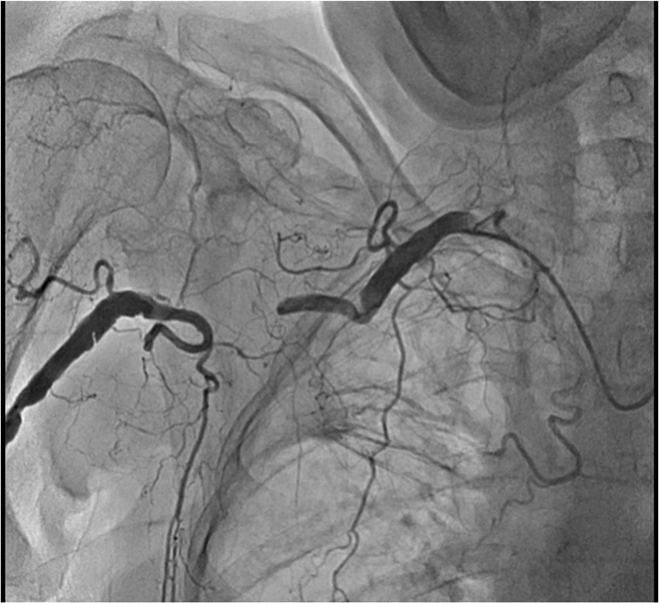
**Peripheral angiogram of occluded right axillary artery with bilateral transradial approach.** Simultaneous injection through JR 4.0 6 F GC from the right radial artery and Simmons 2 5 F diagnostic catheter from the left RA.

**Figure 3:**
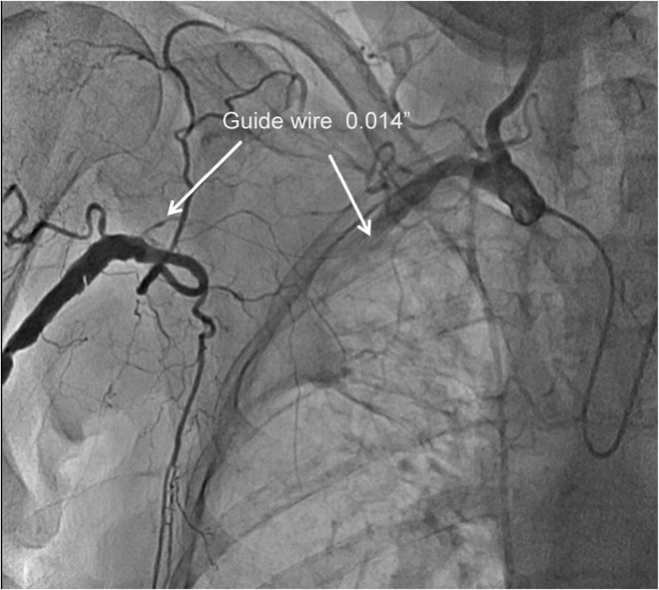
**Crossing the lesion with hydrophilic 0.014? guidewire.**

**Figure 4:**
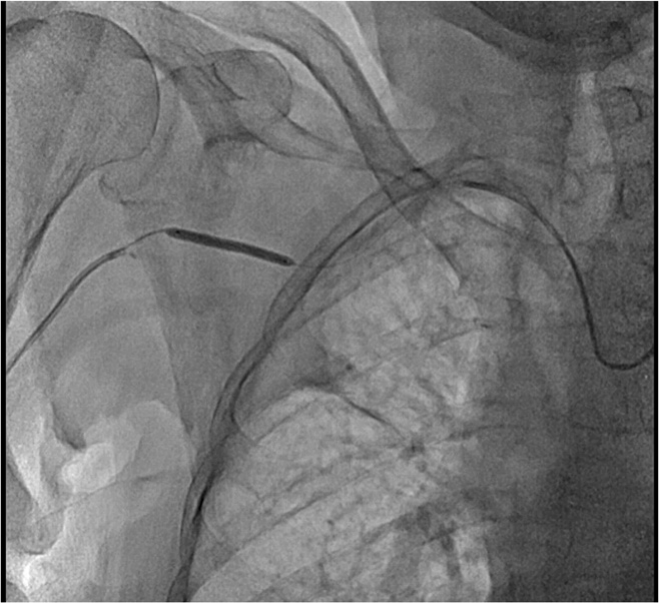
**Predilatation with balloon catheter 3.0/30 mm.**

**Figure 5:**
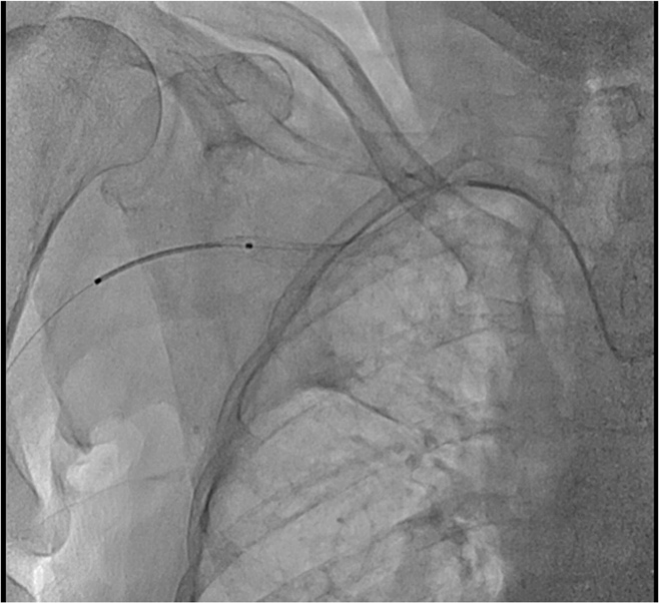
**Implantation of two self-expandable stents Xpert 8.0/40 mm.**

From the right arm we used JR 4.0 6 F guiding catheter (Launcher, Medtronic), and from the left arm Simmons 2 5Fr diagnostic catheter was utilized for contralateral injections. The occlusion and collaterals were documented with simultaneous bilateral injections (Figure 2). Contralateral injection confirmed proper wire advancement within the lesion. The occlusion was successfully crossed with hydrophilic 0.014? wire (HT Pilot 150, Abbott Vascular) (Figure 3). Balloon predilatation was made with balloon catheter 3.00/30 mm, (Sprinter, Medtronic) (Figure 4). Afterward, CTO was recrossed with the 6 F GC in order to exchange the HT Pilot 150 guidewire with heavily supportive 0.014? Iron Man 300 mm guidewire (Abbott Vascular). Two Xpert self-expandable stents 8.0/40 mm (Abbott Vascular) were advanced solely through guidewire without guiding catheter (Figure 5).

**Figure 6:**
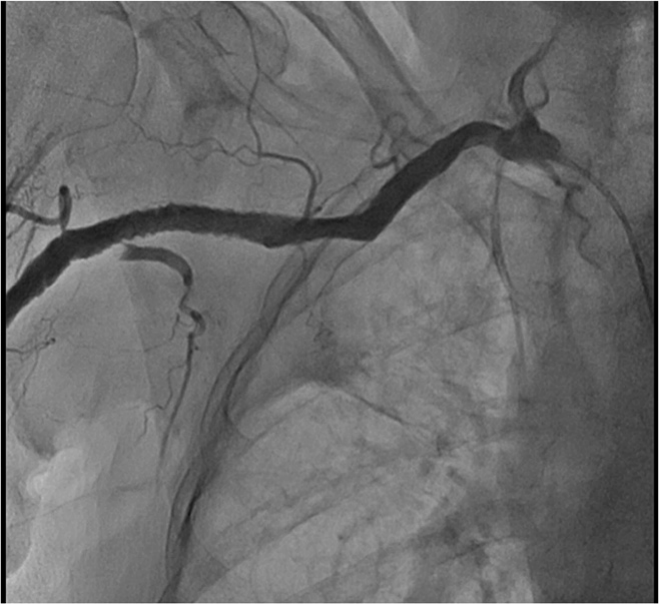
**Final angiography with normal flow through the two stents deployed over the axillary artery occlusion.**

Proper positioning was reconfirmed with contralateral injections through the Simmons 2 catheter. Post dilatation was made with 7.00/30 mm balloon catheter (Viatrac 14 plus, Abbott Vascular) at high pressure. Final angiography showed normal brisk flow through the deployed stents (Figure 6). We applied patent haemostasis on the puncture sites. Total amount of 320 ml of contrast has been used, with fluoroscopy time of 29 min. The day after intervention control duplex-ultrasonography revealed normal and symmetric arterial signals of both arms. The pain in the right arm was relieved after intervention and patient was discharged the following day without any bleeding or vascular complications.

**Figure 7:**
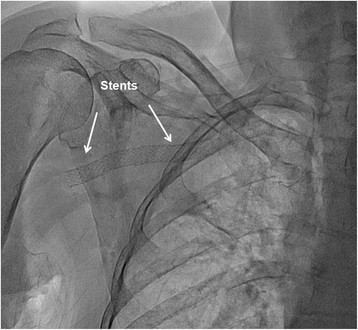
**Two years follow up fluoroscopy.**

**Figure 8:**
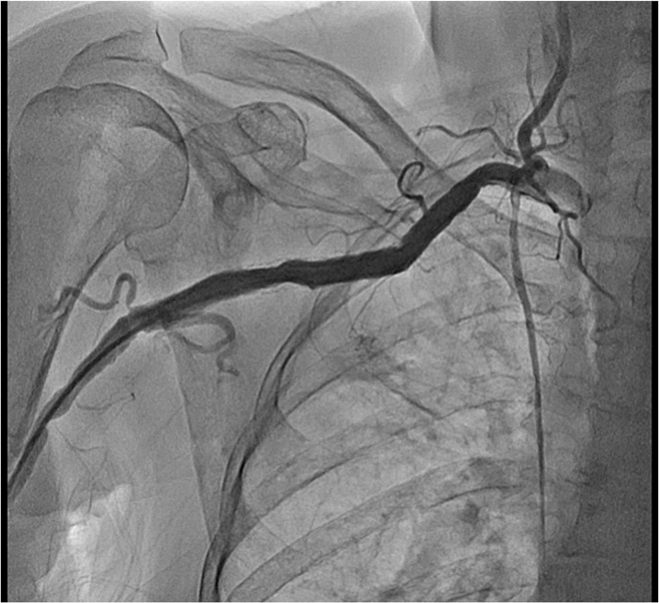
**Two years follow up angiography.**

At two years follow up patient was completely asymptomatic, without any signs of right arm ischemia. Ipsilateral radial and ulnar artery pulsations were normal. Control duplex ultrasonography showed patent stent and normal flow in the right axillary artery. Both stents were widely patent at two years follow up angiography. (Figures 7 and 8).

## Discussion

There are various reasons for axillary artery stenosis as mechanical injuries, Takayasu aoro-arteritis, giant cell arteritis and radiation induced arteritis. However, atherosclerotic axillary artery disease is most commonly met in clinical practice, especially in hemodynamically unstable lesions [1],[2]. Most lesions on the axillary artery in patients are symptomatic [1]. In our case there was dual etiology for the CTO: radiation induced arteritis and atherosclerosis as well [3].

Angioplasty and recanalisation of the CTO lesion in this particular case relieved the disabling symptoms and enabled patient's normal functioning in daily activities.

There are few published cases of stenting of axillary artery [1]-[6], and no data about using bilateral transradial approach. Surgical interventions in these lesions showed higher rate of complications as stroke and TIA and also higher mortality rate [6],[7]. However, percutaneous angioplasty of the axillary artery is associated with less neurological complications [5],[6],[8]. Technically, the most important aspect is to avoid vertebral and common carotid artery take off. In our particular case occlusion was distal to the vertebral artery as well as common carotid artery. The retrograde right radial access allowed wire manipulation without involving takeoffs of supraaortic vessels.

The alternative transfemoral acces would necessitate larger bore guiding catheter (7Fr) and antegrade access of the CTO. Most of the currently available peripheral stents require at least 7 F guiding catheter or long 6 F guiding sheath. In our case we performed axillary artery stenting through short 6 F sheath (10 cm) from the right radial artery. This was only possible with contralateral control from the diagnostic catheter from the left radial access that allowed proper stent positioning. Proximal cap of the CTO is usually harder than the distal end of the CTO. In our case, contralateral injections allowed delivery and proper positioning of two stents only through the 0.014? guidewire; without guiding catheter and without the risk of involving the ipsilateral vertebral and innominate arteries.

Furthermore, transradial approach has been proven to have less bleeding and vascular complications in comparison to transfemoral approach in patients undergoing PCI or endovascular procedures [9]-[11].

## Conclusion

Retrograde approach with bilateral radial access is feasible and safe strategy in recanalisation of the axillary artery chronic total occlusion. Further reports and studies are needed to compare alternative techniques in treating complex axillary artery lesions.

## Consent

Written informed consent was obtained from the patient for publication of this Case report and any accompanying images. A copy of the written consent is available for review by the Editor-in-Chief of this journal.

## Authors' information

SK - is the director of the University Clinic of Cardiology where the patient was treated. He is also a professor at the Ss. Cyril and Methodius University Medical Faculty in Skopje, Macedonia.

## Authors’ original submitted files for images

Below are the links to the authors’ original submitted files for images. Authors’ original file for figure 1
Authors’ original file for figure 2
Authors’ original file for figure 3
Authors’ original file for figure 4
Authors’ original file for figure 5
Authors’ original file for figure 6
Authors’ original file for figure 7
Authors’ original file for figure 8

## Abbreviations

TRA: Transradial approach
CTO: Chronic total occlusion
