# Supplementary figures and images for: Bilateral trans-radial approach in stenting of occluded right axillary artery
Source: J Cardiothorac Surg. 2014 Aug 23;9:138. doi: 10.1186/s13019-014-0138-0 (PMC4164749; doi:10.1186/s13019-014-0138-0)

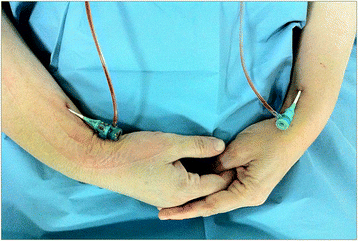

Supplement: Supplementary file 1 — Authors’ original file for figure 1 [file 13019_2014_138_MOESM1_ESM.gif]

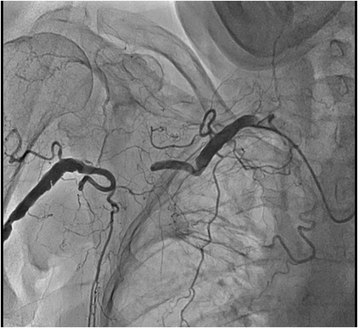

Supplement: Supplementary file 2 — Authors’ original file for figure 2 [file 13019_2014_138_MOESM2_ESM.gif]

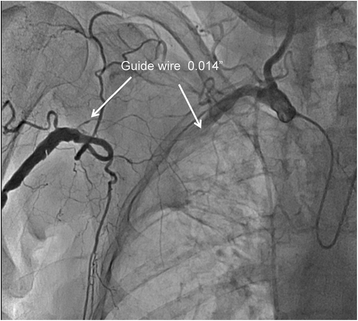

Supplement: Supplementary file 3 — Authors’ original file for figure 3 [file 13019_2014_138_MOESM3_ESM.gif]

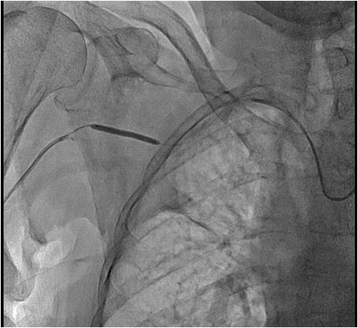

Supplement: Supplementary file 4 — Authors’ original file for figure 4 [file 13019_2014_138_MOESM4_ESM.gif]

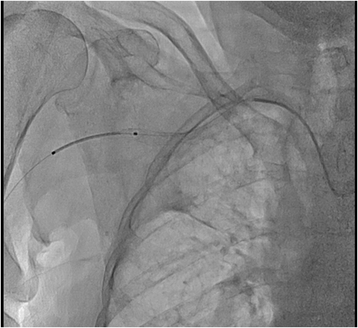

Supplement: Supplementary file 5 — Authors’ original file for figure 5 [file 13019_2014_138_MOESM5_ESM.gif]

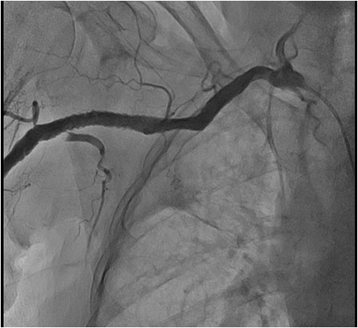

Supplement: Supplementary file 6 — Authors’ original file for figure 6 [file 13019_2014_138_MOESM6_ESM.gif]

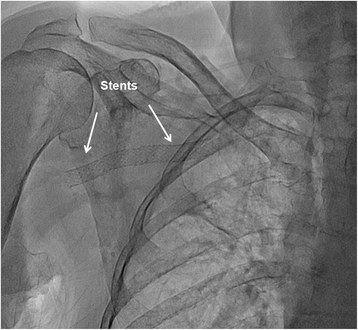

Supplement: Supplementary file 7 — Authors’ original file for figure 7 [file 13019_2014_138_MOESM7_ESM.gif]

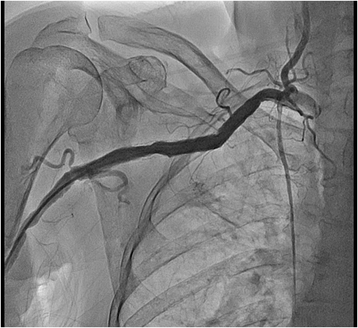

Supplement: Supplementary file 8 — Authors’ original file for figure 8 [file 13019_2014_138_MOESM8_ESM.gif]
